# Changing Patterns of Digestive Disease Mortality in Older Adults in Poland, 2000–2022: Age- and Sex-Specific Trends in Liver Disease Mortality

**DOI:** 10.3390/jcm15187001

**Published:** 2026-09-10

**Authors:** Monika Burzyńska, Małgorzata Pikala

**Affiliations:** 1 Department of Public Health, Epidemiology and Biostatistics, Public Health Observatory, Medical University of Lodz, Żeligowskiego 7/9, 90-752 Lodz, Poland; malgorzata.pikala@umed.lodz.pl; 2 Successful Ageing Research Unit, Department of Internal Medicine and Geriatric Cardiology, Centre of Postgraduate Medical Education, 01-813 Warsaw, Poland

**Keywords:** digestive disease, alcohol-related liver disease, liver cirrhosis, mortality trends, epidemiology, aging

## Abstract

**Background**: The aim of this study was to assess long-term trends in mortality due to digestive diseases among older adults in Poland. **Methods**: Mortality data for the years 2000–2022 were analyzed for individuals aged ≥65 years. Age-standardized death rates (SDRs) were calculated. Particular attention was paid to liver cirrhosis-related mortality, operationalized according to the tenth revision of the International Statistical Classification of Diseases and Related Health Problems (ICD-10) joined categories K70 and K74. Temporal trends were assessed using joinpoint regression to estimate annual percentage change (APC) and average annual percentage change (AAPC) stratified by sex and age groups (65–74 and ≥75 years). The total number of deaths included in the statistical analysis was 6,645,408. **Results**: In early old age (65–74 years), the proportion of deaths due to digestive diseases increased in both men (from 3.6% to 5.0%) and women (from 4.1% to 4.5%), while a decrease was observed in late old age (≥75 years). The combined K70/K74-coded liver disease category represented an important component of digestive disease mortality. Among women aged 65–74 years, a significant upward trend in SDR was observed after 2015 (APC 3.5%, *p* < 0.05), with an increase from 54.7 to 70.8. In men of the same age, SDR remained relatively stable overall (160.5 to 171.6), although mortality due to liver-related diseases increased significantly after 2015 (APC 6.7%, *p* < 0.05). In older age groups, declining trends were observed until the late 2010s, followed by recent non-significant increases. **Conclusions**: Mortality patterns for alcohol-related liver disease and hepatic fibrosis/cirrhosis showed particularly unfavorable recent trends in adults aged 65–74 years. These findings highlight the need for continued surveillance and prevention of chronic liver disease in ageing populations.

## 1. Introduction

Population ageing is one of the most important public health challenges of the 21st century and is associated with substantial changes in patterns of morbidity and mortality [1]. As life expectancy increases, chronic non-communicable diseases account for an increasing proportion of the disease burden among older adults. Among these conditions, diseases of the digestive system represent an important cause of mortality, disability, and healthcare utilization in many European countries, including Poland [2].

Chronic liver diseases constitute a particularly relevant component of the burden of digestive diseases. Liver fibrosis and cirrhosis represent the final common pathway of multiple chronic liver disorders and are associated with substantial mortality worldwide. According to recent Global Burden of Disease estimates, cirrhosis and other chronic liver diseases remain major contributors to premature mortality and disability, with considerable variation across regions and populations. Furthermore, projections suggest that the burden of chronic liver disease may continue to increase over the coming decades, particularly in ageing societies [3].

The etiology of liver fibrosis and cirrhosis is heterogeneous. Alcohol-related liver disease remains one of the leading causes of cirrhosis and liver-related mortality worldwide, especially in Europe [4,5]. However, other causes, including viral hepatitis, metabolic dysfunction-associated steatotic liver disease (MASLD), obesity, type 2 diabetes, and metabolic syndrome, also contribute substantially to the development and progression of chronic liver disease [6,7]. These factors often coexist and interact, making the interpretation of population-level mortality trends increasingly complex.

In recent decades, important changes have occurred in the epidemiology of chronic liver disease. While alcohol consumption continues to represent a major public health concern in Central and Eastern Europe, the increasing prevalence of obesity, diabetes, and metabolic dysfunction has contributed to a growing burden of liver fibrosis and cirrhosis in many countries [5,6,7,8]. Consequently, contemporary mortality patterns associated with chronic liver disease are likely to reflect the combined influence of multiple behavioral, metabolic, and demographic factors rather than a single etiological pathway.

Despite the growing burden of digestive and chronic liver diseases, relatively little is known about long-term mortality trends among older adults in Poland. Previous studies have mainly focused on the overall population or selected causes of death, whereas evidence on sex-specific mortality patterns among adults aged 65 years and older remains limited. Previous findings suggest that mortality patterns may differ substantially according to age and sex, indicating that important shifts in disease burden may be occurring within specific elderly subpopulations. In particular, it remains unclear whether mortality trends differ between early old age (65–74 years) and late old age (≥75 years). A better understanding of these patterns is essential for identifying vulnerable groups and informing public health strategies aimed at reducing avoidable mortality in an ageing society.

Therefore, the aim of the present study was to assess long-term trends in mortality due to digestive diseases among older adults in Poland between 2000 and 2022, with particular emphasis on age- and sex-specific patterns and mortality associated with K70/K74-coded liver disease.

## 2. Materials and Methods

The study material consisted of a database including information on all deaths among Polish residents aged 65 years and older between 2000 and 2022, obtained from Statistics Poland. The analysed data include all registered deaths, irrespective of place of death (hospital, home, or other settings), as recorded in the national death registry. For each death, the underlying cause of death, coded according to the International Classification of Diseases, 10th Revision (ICD-10), was used for cause-specific mortality analyses. The total number of deaths included in the statistical analysis was 6,645,408.

Deaths due to digestive system diseases were identified using ICD-10 codes K00–K93. Particular attention was paid to the most common groups of causes within this category: liver cirrhosis-related mortality, operationalized according to the ICD-10 categories K70 and K74, corresponding to alcohol-related liver disease and hepatic fibrosis/cirrhosis, respectively. Mortality was analyzed separately for women and men and for two age groups: 65–74 years and ≥75 years. The 65–74-year group was used to represent early old age, whereas individuals aged ≥75 years were considered to represent late old age. This categorization was selected to distinguish mortality patterns in the younger segment of the older population from those occurring at more advanced ages, where multimorbidity, frailty and competing causes of death are more prevalent. To assess changes in the structure of deaths, proportional mortality ratios were calculated for the first and the last year of the study period. Mortality rates were calculated as the number of deaths divided by the corresponding population at risk and expressed per 100,000 population. Age-standardized death rates (SDRs) were calculated using the direct standardization method and the 2012 revision of the European Standard Population [9], using the age-specific population estimates for each calendar year obtained from Statistics Poland. The use of standardized rates allowed comparison of mortality trends over time while minimizing the influence of changes in the age structure of the Polish population during the study period. Temporal trends in SDRs were analyzed using the Joinpoint Regression Program (version 3.5.2; National Cancer Institute, Bethesda, MD, USA), developed by the U.S. National Cancer Institute within the Surveillance, Epidemiology, and End Results (SEER) Program [10]. Log-linear joinpoint regression models were fitted to annual SDRs to identify significant changes in trend slopes over time.

The analysis allowed a maximum of 4 joinpoints, with a minimum of 3 observations between joinpoints and at the beginning and end of the study period. Model selection was based on the Monte Carlo permutation test implemented in the Joinpoint Regression Program. The statistical significance level was set at *p* = 0.05. For each identified segment, the annual percentage change (APC) and corresponding 95% confidence interval (CI) were calculated. The average annual percentage change (AAPC) was calculated for the entire study period as a summary measure of the overall temporal trend. An APC or AAPC was considered statistically significant when its 95% CI did not include zero.

The study was approved by the Bioethics Committee of the Medical University of Lodz, No. RNN/422/12/KB. Written informed consent for participation was not required for this study in accordance with the national legislation and the institutional requirements.

## 3. Results

The most common categories of causes of death among individuals aged 65 and over in Poland were, in order: cardiovascular diseases, malignant neoplasms, and respiratory diseases. Digestive system diseases ranked fourth among the leading causes of death. For this category, an increase in the proportion of deaths was observed in both men and women in early old age, while a decrease was noted in both sexes in late old age. Among men aged 65–74 years, the proportion of deaths due to digestive diseases increased from 3.6% in 2000 to 5.0% in 2022, whereas in those aged 75+ it decreased from 2.9% to 2.5%. In women, the proportion of deaths in early old age rose from 4.1% to 4.5%, while in late old age it declined from 3.0% to 2.8%.

Within digestive system diseases, the combined K70/K74-coded liver disease category represented an important contributor to mortality in the analyzed age and sex groups. For this combined category, an increase in the proportion of deaths was observed across all analyzed age and sex groups.

**Figure 1:**
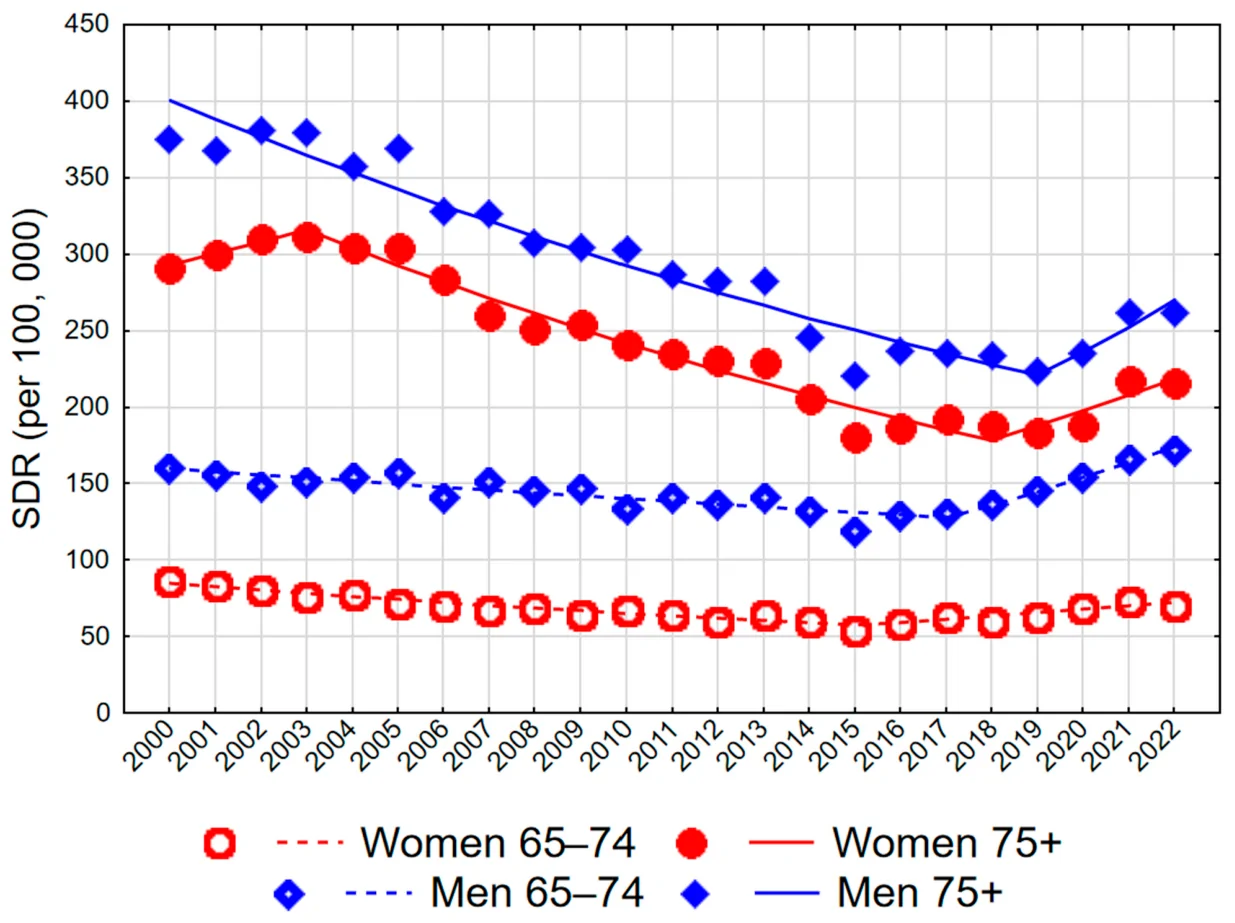
Trends in age-standardized death rates (SDR, per 100,000 population) due to digestive diseases in Poland, 2000–2022, by sex and age group (65–74 years and ≥75 years).

However, analyses based on age-standardized death rates revealed a more complex pattern. Recent numerical increases in mortality were observed in several subgroups, although not all increases reached statistical significance. Among women in early old age, an increase in the standardized death rate (SDR) began in 2015, following a prior decline between 2000 and 2015 at an annual rate of −2.6% (*p* < 0.05). Between 2015 and 2022, the annual percentage change (APC) was 3.5% (*p* < 0.05), resulting in an increase in SDR from 54.7 to 70.8 (Figure 1, Table 1).

Within digestive system diseases, mortality associated with the combined K70/K74 category represented an important component of the observed mortality burden. Among women aged 65–74 years, after statistically non-significant changes observed between 2000 and 2015, mortality began to increase from 2015 onwards at an annual rate of 5.6% (*p* < 0.05), reaching an SDR of 25.6 in 2022.

**Figure 2:**
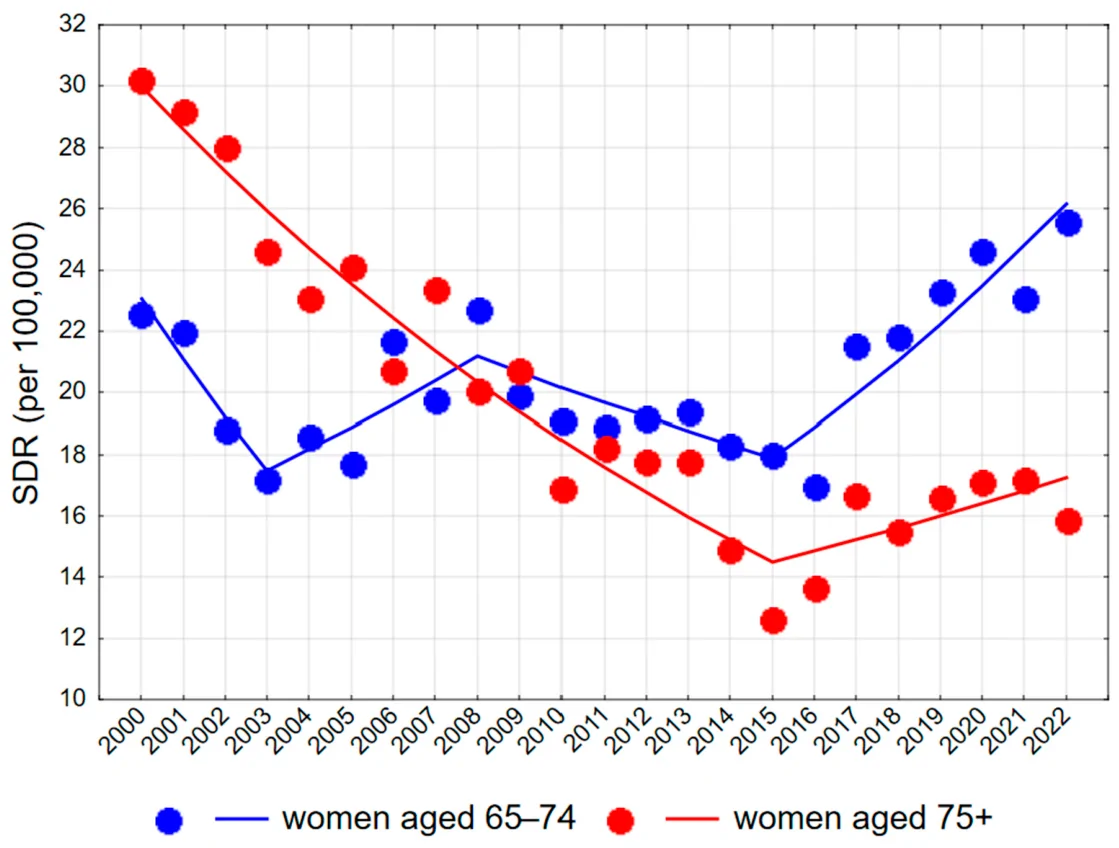
Trends in age-standardized death rates (SDR, per 100,000 population) due to alcohol-related liver disease and hepatic fibrosis and cirrhosis (ICD-10 codes K70 and K74) among women in Poland, 2000–2022.

Regarding mortality due to digestive diseases overall, in women aged 75 years and over, following statistically non-significant changes in SDR between 2000 and 2003, a decline was observed in the period 2003–2018 (APC = −3.7%, *p* < 0.05), followed by an increase between 2018 and 2022 at an annual rate of 5.2% (*p* < 0.05), reaching a value of 216.5 in 2022 (Table 1). Analysis of mortality rates due to alcohol-related liver disease and liver fibrosis and cirrhosis in this group showed a statistically significant decrease in SDR between 2000 and 2015 (APC = −4.7%, *p* < 0.05), followed by a non-significant increase after 2015 (Figure 2).

Among men aged 65–74 years, a statistically significant decline in SDR due to digestive diseases was observed between 2000 and 2017 (APC = −1.3%, *p* < 0.05), followed by a significant increase thereafter (APC = 6.4%, *p* < 0.05) (Table 1, Figure 1). As a result of these opposing trends, SDR changed only slightly, from 160.5 in 2000 to 171.6 in 2022 (AAPC = 0.4%, *p* > 0.05).

**Figure 3:**
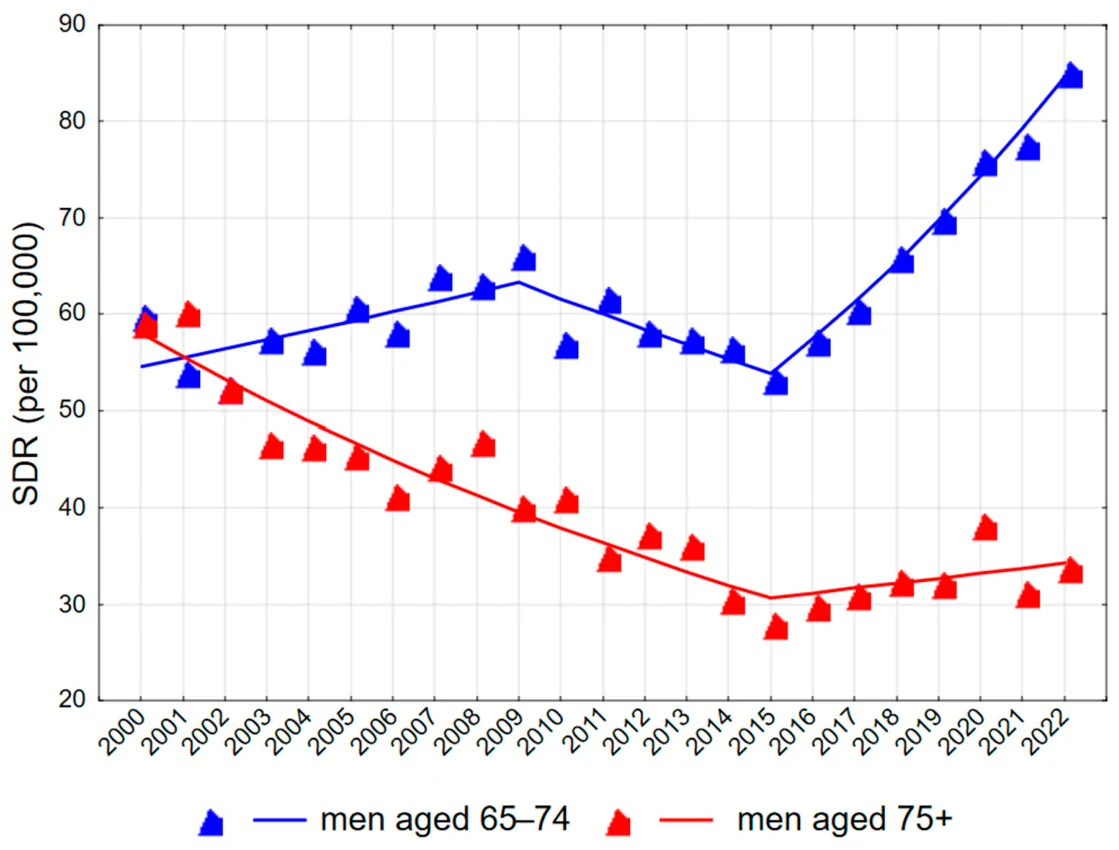
Trends in age-standardized death rates (SDR, per 100,000 population) due to alcohol-related liver disease and hepatic fibrosis and cirrhosis (ICD-10 codes K70 and K74) among men in Poland, 2000–2022.

Trends in mortality due to alcohol-related liver disease and liver fibrosis and cirrhosis in this male age group showed a different pattern. Two changes in trend were identified: an increase in SDR between 2000 and 2009 (APC = 1.7%, *p* < 0.05), followed by non-significant changes between 2009 and 2015, and a subsequent increase between 2015 and 2022 at an annual rate of 6.7% (*p* < 0.05). Consequently, SDR increased from 59.8 in 2000 to 85.0 in 2022 (Figure 3, Table 1).

Among the oldest men (aged 75+), mortality due to digestive diseases decreased between 2000 and 2019 (APC = −3.1%, *p* < 0.05), followed by a non-significant increase in the period 2019–2022 (Figure 1, Table 1). A similar pattern was observed for mortality due to alcohol-related liver disease and liver fibrosis and cirrhosis, with a decreasing trend between 2000 and 2015 (APC = −4.2%, *p* < 0.05), followed by a non-significant increase thereafter (Figure 3).

## 4. Discussion

The present study demonstrated divergent long-term patterns in mortality from diseases of the digestive system among individuals aged 65 years and older in Poland. Although digestive diseases remained the fourth leading group of causes of death, their contribution to mortality increased among individuals aged 65–74 years, whereas a more favorable or stable pattern was observed among those aged 75 years and older. The most pronounced recent changes concerned the combined K70/K74 category, comprising alcohol-related liver disease and hepatic fibrosis and cirrhosis. Importantly, this combined category represents a heterogeneous group of liver diseases and should not be interpreted as exclusively alcohol-attributable mortality. The observed findings therefore indicate a changing burden of chronic liver disease and cirrhosis in older adults rather than providing evidence for a single etiological mechanism.

A key finding of the present study was the marked divergence in mortality trends between early old age (65–74 years) and late old age (≥75 years). Among individuals aged 65–74 years, mortality from digestive diseases increased in the later part of the observation period in both sexes, with particularly pronounced recent increases among men. In contrast, among individuals aged ≥75 years, overall mortality from digestive diseases declined over earlier periods, followed by more recent increases that were not consistently statistically significant. A similar age-specific pattern was observed for the combined K70/K74 category, for which the most pronounced recent increases occurred among individuals aged 65–74 years.

This age-specific divergence is important from a public health perspective, as it suggests that the burden of digestive and chronic liver diseases is not distributed uniformly across older populations. Individuals aged 65–74 years may represent a particularly relevant population for prevention and early detection strategies. At the same time, the more stable mortality observed among individuals aged ≥75 years should not necessarily be interpreted as evidence of a lower prevalence or clinical importance of chronic liver disease in this age group.

The observed findings are consistent with the broader epidemiological evidence indicating that chronic liver disease remains an important cause of morbidity and mortality worldwide. Global analyses have demonstrated a substantial burden of liver cirrhosis and continuing changes in its epidemiology [3,6]. Alcohol consumption remains an important risk factor for liver cirrhosis and alcohol-associated liver disease, and its contribution to digestive disease mortality is particularly relevant in Europe [4,5,7]. However, the present study cannot determine the contribution of alcohol to the observed trends in the combined K70/K74 category. The interpretation of the K70/K74 category requires particular caution. K70 specifically identifies alcohol-related liver disease, whereas K74 includes hepatic fibrosis and cirrhosis irrespective of the underlying etiology. The latter may result from alcohol exposure, metabolic dysfunction-associated steatotic liver disease (MASLD), chronic viral hepatitis, autoimmune liver disease and other chronic hepatic conditions [6,8,11]. Consequently, the combined K70/K74 category used in the present analysis should be regarded as an operational epidemiological category comprising alcohol-related liver disease and hepatic fibrosis/cirrhosis, rather than as a measure of alcohol-attributable mortality alone.

This distinction is particularly relevant when interpreting the increase observed after 2015 among individuals aged 65–74 years. The present analysis demonstrates that mortality within the combined K70/K74 category increased during the later study period; however, the available data do not allow us to determine whether this increase was driven predominantly by alcohol-related disease, metabolic liver disease, viral hepatitis or other causes of hepatic fibrosis and cirrhosis. The observed increase should therefore be interpreted as evidence of an increasing mortality burden associated with chronic liver disease and cirrhosis, with alcohol representing one potentially important contributor.

Alcohol nevertheless provides an important epidemiological context for the present findings. Previous research has established alcohol as a major determinant of liver cirrhosis and alcohol-associated liver disease [4,5,7]. In Poland, alcohol consumption has changed substantially over the past decades, and population-level indicators indicate a persistent burden of alcohol-attributable harm [12,13]. European data similarly demonstrate a substantial contribution of alcohol to liver disease mortality [14]. These observations support the relevance of alcohol-related disease within the K70 component of our analysis, but they cannot establish that alcohol was responsible for the increase observed in the combined K70/K74 category.

Additional information from the PolSenior2 study provides context regarding liver disease and alcohol consumption among older adults in Poland. However, these data cannot be used to determine the etiological contribution of alcohol to K74-coded mortality in the present study [15].

The epidemiology of chronic liver disease has also changed because of the increasing importance of metabolic risk factors. MASLD is strongly associated with obesity, type 2 diabetes and metabolic dysfunction and represents an increasingly important cause of hepatic fibrosis and cirrhosis [8,11]. The earlier literature described these conditions using the terms NAFLD and NASH [16,17], whereas current terminology has shifted toward MASLD. The coexistence of metabolic risk factors and alcohol exposure further complicates the attribution of liver disease to a single cause. Therefore, the mortality patterns observed in the present study may reflect overlapping metabolic, alcohol-related, viral and other etiological pathways.

Several hypotheses may help explain the age-specific patterns observed in our study. The more pronounced increase among individuals aged 65–74 years may be compatible with differences in cumulative exposure over the life course. Historical patterns of alcohol consumption may be one possible contributor. Data from the Standardized European Alcohol Survey (RARHA SEAS) demonstrated differences in drinking patterns among older European populations and provide relevant background for interpreting historical exposure patterns in older adults [18]. However, the present study did not include individual-level alcohol consumption data, and therefore these observations cannot be used to demonstrate that a specific birth cohort was responsible for the observed mortality increase.

In particular, the term “cohort effect” should be interpreted cautiously. Joinpoint regression identifies changes in mortality trends according to calendar time but does not disentangle age, period and birth-cohort effects. Consequently, the present analysis cannot establish that the increase observed after 2015 represents a “baby-boomer cohort effect”. The observed pattern may be compatible with a cohort-related explanation, but it may also reflect calendar-period changes, differences in healthcare, diagnostic practices, population exposures or other factors. Formal age–period–cohort modelling would be required to distinguish these mechanisms.

The differences between early and late old age may also reflect survivor effects and competing risks of death. Individuals who survive to ≥75 years represent a selected subgroup of the population and may differ from younger older adults with respect to cumulative exposure, biological susceptibility, comorbidity and healthcare utilization. Individuals with severe chronic disease may die before reaching advanced age, potentially contributing to lower subsequent mortality from selected causes. In addition, cardiovascular disease, malignant neoplasms, respiratory disease and other conditions account for a substantial proportion of deaths at advanced ages. Consequently, competing causes of death may contribute to the different mortality patterns observed between individuals aged 65–74 and those aged ≥75 years.

These explanations remain hypothetical in the context of the present ecological analysis. The available mortality database does not contain sufficient individual-level information to directly evaluate cumulative alcohol exposure, metabolic health, survivor selection, healthcare utilization or competing-risk mechanisms.

The present findings are broadly consistent with international evidence demonstrating increases in liver-related mortality over recent decades, including among older adults in the United States [19,20]. However, because these studies focused specifically on alcohol-related liver disease, their findings cannot be directly extrapolated to the heterogeneous K70/K74 category examined in the present study.

Studies from Lithuania and Russia suggest that alcohol-related mortality may be influenced by alcohol-control policies [21,22]. However, because the present study did not evaluate policy interventions or individual alcohol exposure, these observations should be regarded as contextual rather than causal explanations for the trends observed in Poland.

The COVID-19 pandemic represents another potential contextual factor affecting the most recent years of observation. Modelling studies have suggested that increased alcohol consumption during the pandemic could contribute to an additional burden of alcohol-associated liver disease [23]. However, the present study was not designed to isolate the effect of the pandemic, and no individual-level information on alcohol consumption, healthcare utilization or COVID-19 status was available. Therefore, the contribution of pandemic-related changes to the mortality patterns observed in 2020–2022 cannot be quantified.

The increasing burden of chronic liver disease mortality represented by the combined K70/K74 category among older adults highlights the need for targeted preventive strategies, including improved screening for harmful alcohol use, early detection of liver disease, and strengthened alcohol control policies. Given projections indicating that mortality from alcohol-related liver disease may increase by up to 75% by 2040, and that the incidence of liver cirrhosis and liver cancer may rise by more than 65% [24], urgent action is required to reverse these unfavorable trends. At the same time, addressing metabolic risk factors and improving integrated care for older adults may be essential to reduce the future burden of digestive diseases [25].

### Strengths and Limitations

This study has several strengths. First, it was based on a large national mortality dataset covering all registered deaths among Polish residents aged 65 years and older over a 23-year period. Second, the analysis allowed long-term trends to be examined separately by sex and age group, thereby identifying differences between early and late old age. Third, the use of standardized mortality rates and joinpoint regression enabled assessment of temporal changes while accounting for changes in population age structure. However, several limitations should be considered. First, the ecological design precludes individual-level causal inference. Second, the database did not contain information on individual alcohol consumption, obesity, diabetes, metabolic dysfunction-associated steatotic liver disease, smoking, socioeconomic status or other potential determinants of liver disease mortality. Third, K74 comprises hepatic fibrosis and cirrhosis of heterogeneous etiologies; therefore, the combined K70/K74 category cannot be interpreted as exclusively alcohol-attributable mortality. Fourth, cause-of-death analyses are susceptible to diagnostic and coding misclassification, and changes in death certification practices over the 23-year period may have influenced temporal comparisons. Fifth, competing causes of death may be particularly relevant in adults aged ≥75 years. Finally, the COVID-19 pandemic may have affected mortality patterns in the final years of observation, although its independent contribution cannot be quantified using the available data.

## 5. Conclusions

In this nationwide population-based study, mortality from digestive diseases showed divergent temporal patterns according to age and sex among older adults in Poland. The most pronounced recent increases were observed among individuals aged 65–74 years, including significant increases in mortality associated with the combined K70/K74 category of alcohol-related liver disease and hepatic fibrosis/cirrhosis. In contrast, recent increases among adults aged ≥75 years were not statistically significant. The findings highlight the changing burden of chronic liver disease in an ageing population and support the need for continued surveillance and prevention addressing alcohol-related as well as metabolic and other causes of liver fibrosis and cirrhosis.

## Figures and Tables

**Table 1:** Time trends of standardized death rates (SDR) for the leading causes of death within digestive system diseases among women and men aged 65–74 years and 75 years and older in Poland, 2000–2022—a joinpoint regression analysis.

Causes of Death	Number of Joinpoints	Years	APC (95% CI)	AAPC (95% CI)
	Women aged 65–74			
Diseases of the digestive system (K00–K93):	1	2000–2015	−2.6 * (−3.0; −2.2)	−0.7 * (−1.2; −0.2)
		2015–2022	3.5 * (2.1; 4.8)	
Alcohol-related liver disease and fibrosis and cirrhosis of liver (K70, K74)	3	2000–2003	−8.9 (−17.4; 0.4)	0.6 (−1.5; 2.7)
		2003–2008	3.9 (−2.3; 10.5)	
		2008–2015	−2.4 (−5.6; 0.9)	
		2015–2022	5.6 * (2.9; 8.4)	
	Women aged 75+			
Diseases of the digestive system (K00–K93):	2	2000–2003	2.6 (−3.8; 9.3)	−1.3 * (−2.4; −0.2)
		2003–2018	−3.7 * (−4.3; −3.2)	
		2018–2022	5.2 * (1.0; 9.5)	
Alcohol-related liver disease and fibrosis and cirrhosis of liver (K70, K74)	1	2000–2015	−4.7 * (−5.5; −4.0)	−2.5 * (−3.4; −1.6)
		2015–2022	2.5 (−0.1; 5.2)	
	Men aged 65–74			
Diseases of the digestive system (K00–K93):	1	2000–2017	−1.3 * (−1.7; −0.9)	−0.4 (−1.1; 0.3)
		2017–2022	6.4 * (3.7; 9.1)	
Alcohol-related liver disease and fibrosis and cirrhosis of liver (K70, K74)	2	2000–2009	1.7 * (0.5; 2.8)	−0.7 (−1.8; 0.4)
		2009–2015	−2.7 (−5.3; 0.1)	
		2015–2022	6.7 * (4.9; 8.5)	
	Men aged 75+			
Diseases of the digestive system (K00–K93):	1	2000–2019	−3.1 * (−3.5; −2.5)	−2.4 * (−3.3; −1.4)
		2019–2022	6.9 (−0.6; 14.9)	
Alcohol-related liver disease and fibrosis and cirrhosis of liver (K70, K74)	1	2000–2015	−4.2 * (−5.0; −3.3)	−0.8 * (−1.6; 0.0)
		2015–2022	1.6 (−1.2; 4.5)	

Abbreviations: SDR, age-standardized death rate (per 100,000 population); APC, annual percentage change; AAPC, average annual percentage change; CI, confidence interval. * Statistically significant at *p* < 0.05.

## Data Availability

The data presented in this study are available on request from the corresponding author.
